# A Single Nucleotide Polymorphism within the Acetyl-Coenzyme A Carboxylase Beta Gene Is Associated with Proteinuria in Patients with Type 2 Diabetes

**DOI:** 10.1371/journal.pgen.1000842

**Published:** 2010-02-12

**Authors:** Shiro Maeda, Masa-aki Kobayashi, Shin-ichi Araki, Tetsuya Babazono, Barry I. Freedman, Meredith A. Bostrom, Jessica N. Cooke, Masao Toyoda, Tomoya Umezono, Lise Tarnow, Torben Hansen, Peter Gaede, Anders Jorsal, Daniel P. K. Ng, Minoru Ikeda, Toru Yanagimoto, Tatsuhiko Tsunoda, Hiroyuki Unoki, Koichi Kawai, Masahito Imanishi, Daisuke Suzuki, Hyoung Doo Shin, Kyong Soo Park, Atsunori Kashiwagi, Yasuhiko Iwamoto, Kohei Kaku, Ryuzo Kawamori, Hans-Henrik Parving, Donald W. Bowden, Oluf Pedersen, Yusuke Nakamura

**Affiliations:** 1 Laboratory for Endocrinology and Metabolism, RIKEN Center for Genomic Medicine, Yokohama, Kanagawa, Japan; 2 Discovery Research Laboratories, Shionogi & Co., Toyonaka, Osaka, Japan; 3 Department of Medicine, Shiga University of Medical Science, Otsu, Shiga, Japan; 4 The Diabetes Center, Tokyo Women's Medical University, Tokyo, Japan; 5 Wake Forest University School of Medicine, Winston-Salem, North Carolina, United States of America; 6 Division of Nephrology and Metabolism, Department of Internal Medicine, Tokai University School of Medicine, Isehara, Kanagawa, Japan; 7 Steno Diabetes Center and Hagedorn Research Institute, Gentofte, Denmark; 8 Faculty of Health Sciences, University of Southern Denmark, Odense, Denmark; 9 Department of Epidemiology and Public Health, National University of Singapore, Singapore; 10 Developmental Research Laboratories, Shionogi & Co., Toyonaka, Osaka, Japan; 11 Laboratory for Medical Informatics, RIKEN Center for Genomic Medicine, Yokohama, Kanagawa, Japan; 12 Kawai Clinic, Tsukuba, Ibaraki, Japan; 13 Division of Nephrology and Hypertension, Department of Internal Medicine, Osaka City General Hospital, Osaka, Japan; 14 Department of Life Science, Sogang University, Seoul, Korea; 15 Department of Molecular Medicine and Biopharmaceutical Sciences, Graduate School of Convergence Science and Technology, Seoul National University, Seoul, Korea; 16 Department of Internal Medicine, College of Medicine, Seoul National University, Seoul, Korea; 17 Division of Endocrinology and Metabolism, Department of Internal Medicine, Kawasaki Medical School, Kurashiki, Okayama, Japan; 18 Department of Medicine, Metabolism, and Endocrinology, School of Medicine, Juntendo University, Tokyo, Japan; 19 Faculty of Health Sciences, University of Aarhus, Aarhus, Denmark; 20 Department of Medical Endocrinology, Rigshospitalet, Copenhagen, Denmark; 21 Institute of Biomedical Sciences, Faculty of Health Sciences, University of Copenhagen, Copenhagen, Denmark; 22 Laboratory of Molecular Medicine, Human Genome Center, Institute of Medical Science, University of Tokyo, Tokyo, Japan; University of Oxford, United Kingdom

## Abstract

It has been suggested that genetic susceptibility plays an important role in the pathogenesis of diabetic nephropathy. A large-scale genotyping analysis of gene-based single nucleotide polymorphisms (SNPs) in Japanese patients with type 2 diabetes identified the gene encoding acetyl-coenzyme A carboxylase beta (*ACACB*) as a candidate for a susceptibility to diabetic nephropathy; the landmark SNP was found in the intron 18 of *ACACB* (rs2268388: intron 18 +4139 C > T, p = 1.4×10^−6^, odds ratio = 1.61, 95% confidence interval [CI]: 1.33–1.96). The association of this SNP with diabetic nephropathy was examined in 9 independent studies (4 from Japan including the original study, one Singaporean, one Korean, and two European) with type 2 diabetes. One case-control study involving European patients with type 1 diabetes was included. The frequency of the T allele for SNP rs2268388 was consistently higher among patients with type 2 diabetes and proteinuria. A meta-analysis revealed that rs2268388 was significantly associated with proteinuria in Japanese patients with type 2 diabetes (p = 5.35×10^−8^, odds ratio = 1.61, 95% Cl: 1.35–1.91). Rs2268388 was also associated with type 2 diabetes–associated end-stage renal disease (ESRD) in European Americans (p = 6×10^−4^, odds ratio = 1.61, 95% Cl: 1.22–2.13). Significant association was not detected between this SNP and nephropathy in those with type 1 diabetes. A subsequent *in vitro* functional analysis revealed that a 29-bp DNA fragment, including rs2268388, had significant enhancer activity in cultured human renal proximal tubular epithelial cells. Fragments corresponding to the disease susceptibility allele (T) had higher enhancer activity than those of the major allele. These results suggest that *ACACB* is a strong candidate for conferring susceptibility for proteinuria in patients with type 2 diabetes.

## Author Summary

Although cumulative epidemiological findings have suggested that genetic susceptibility plays an important role in the pathogenesis of diabetic nephropathy, no gene conferring susceptibility to diabetic nephropathy has been definitively identified. In a large-scale association study of 1,312 Japanese subjects with type 2 diabetes using SNPs from a Japanese SNP database, we show that the T-allele of *ACACB* rs2268388 is associated with diabetic nephropathy. We also show that the association is consistently observed in patients with type 2 diabetes and proteinuria across different ethnic groups, including populations of European descent. Because a DNA fragment corresponding to the disease susceptibility allele is shown to have higher enhancer activity, we hypothesize that the increase in the expression and/or activity of the encoded acetyl-coenzyme A carboxylase beta contributes to the development and progression of diabetic nephropathy. Our present analysis provides novel insight into the pathogenesis of diabetic nephropathy. This finding is important because diabetic nephropathy is a leading cause of end-stage renal disease and affects life expectancy in subjects with type 2 diabetes.

## Introduction

Diabetic nephropathy is a leading cause of end-stage renal disease (ESRD) in Western countries [1] and in Japan [2]. The rising incidence of diabetic nephropathy, especially among patients with type 2 diabetes, is a serious worldwide concern in terms of both poor prognosis and medical costs. The pathogenesis of diabetic nephropathy has not been fully elucidated. However, susceptibility to diabetic nephropathy appears to be determined by multiple genetic and environmental risk factors, and genetic susceptibility plays an important role in its development and progression [3],[4].

Both candidate gene approaches and genome-wide linkage analyses have suggested several candidate genes with potential impact on diabetic nephropathy. However, these findings have not been robustly replicated [5],[6], and many susceptibility genes for diabetic nephropathy remain to be identified. The recent development of single nucleotide polymorphism (SNP) typing technology and insights into patterns of linkage disequilibrium (LD) in the human genome have facilitated genome-wide association studies (GWASs) for investigating genes associated with disease susceptibility across the entire human genome. GWASs conducted by several independent research groups in Europe, United States [7],[8] and Japan [9],[10] have identified multiple loci associated with susceptibility to common complex traits, including type 2 diabetes. Recently conducted GWAS in a population of European descent identified 4 distinct loci associated with diabetic nephropathy in type 1 diabetes. Two of these loci were replicated in a population of the Diabetes Control and Complications Trial (DCCT)/Epidemiology of Diabetes Interventions and Complications (EDIC) cohorts [11].

With the aim of identifying loci involved in susceptibility to common diseases, we initiated a large-scale association study using SNPs from a Japanese SNP database (JSNP: http://snp.ims.u-tokyo.ac.jp/) [12],[13], that was established before creation of the HapMap database. Through this project, we have previously identified genes encoding solute carrier family 12 (sodium/chloride) member 3 (*SLC12A3*: MIM 600968, Online Mendelian Inheritance in Man: http://www.ncbi.nlm.nih.gov/omim)[14], engulfment and cell motility 1 (*ELMO1*: MIM 606420)[15], and neurocalcin δ (*NCALD*: MIM 606722)[16] as being associated with susceptibility to diabetic nephropathy. The *ELMO1* association has been replicated in African Americans [17] and European Americans [18].

In the present study, we extended a previous large-scale association study for diabetic nephropathy, and provide evidence that a SNP within the acetyl-coenzyme A (CoA) carboxylase beta gene (*ACACB*; MIM: 601557) contributes to an increased prevalence of proteinuria in patients with type 2 diabetes across different ethnic populations.

## Results

We extended our prior analysis to SNPs with p values between 0.01 and 0.05, and examined the association of these SNPs with diabetic nephropathy in a larger study sample. In this analysis, a SNP within *ACACB* showed the strongest association with diabetic nephropathy in Japanese patients with type 2 diabetes (rs2268388: intron 18 +4139 C > T, p = 1.4×10^−6^, odds ratio [OR]  = 1.61, 95% confidence interval [Cl]: 1.33–1.96, Table 1).

**Table 1:** Top 4 SNPs associated with diabetic nephropathy in a genome-wide screening.

	Nephropathy(a)	p for HWE(b)	Control(a)	p for HWE(b)	Smallest p (model)
rs2268388 (C > T)	0.25 (413/276/48)	0.83	0.17 (379/155/18)	0.66	1.4×10^−6^ (allelic)
Ch 12, *ACACB*					
rs2250736 (C > T)	0.278 (394/288/63)	0.32	0.338 (233/262/55)	0.13	0.0002 (dominant)
Ch 3, *CACNA1D*					
rs3777587 (G > A)	0.523 (156/399/191)	0.05	0.459 (166/269/120)	0.57	0.0002 (dominant)
Ch 6, *CLIC5*					
rs7148607 (T > C)	0.34 (318/353/76)	0.06	0.39 (219/243/94)	0.13	0.0003 (recessive)
Ch 14					

**^(a)^**minor allele frequencies are shown, and genotype counts are in parenthesis.

**^(b)^**p values for Hardy Weinberg equilibrium test.

**Figure 1:**
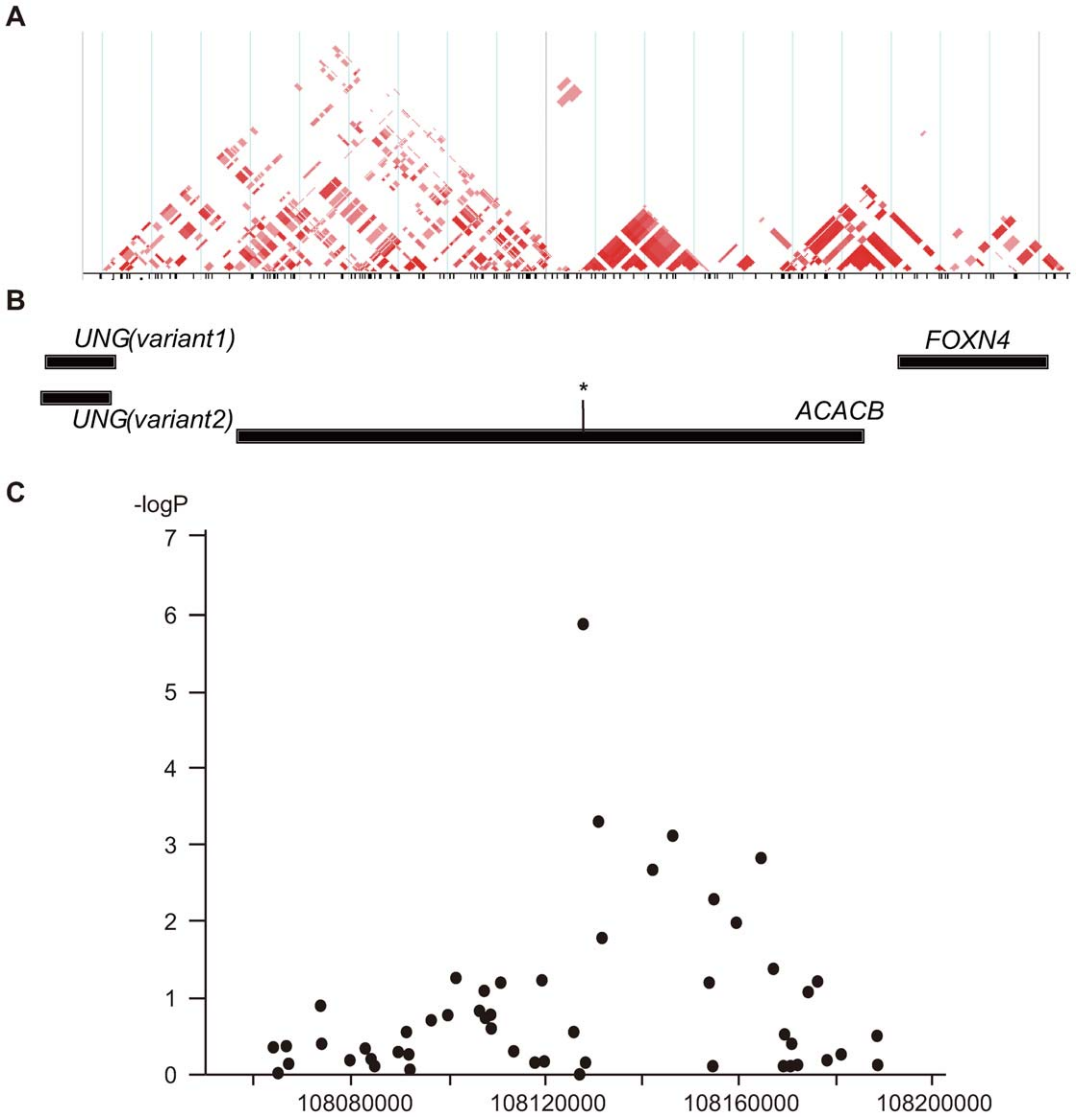
Schematic view of the association of SNPs in the *ACACB* region with diabetic nephropathy. (A) Pairwise correlation structure at a 200-kb interval around SNP rs2268338 analyzed by Haploview (Haploview: http://www.broadinstitute.org/haploview/haploview). The plot includes pairwise r^2^ values from the HapMap release 24 for the JPT population. (B) Genes located at this locus. The asterisk indicates the SNP rs2268388 at intron 18 of the *ACACB*. (C) Results of a case-control association study for diabetic nephropathy in 754 Japanese individuals with type 2 diabetes having overt proteinuria and 558 control individuals with type 2 diabetes and normoalbuminuria. The log_10_-transformed p values for an additive model are plotted on the Y-axis. The X-axis indicates chromosomal position at this locus.

Subsequent LD mapping around this region with data for 264 SNPs with allele frequencies ≥0.1 from HapMap database (HapMap: http://hapmap.ncbi.nlm.nih.gov/) for the Japanese, identified a 20-kb LD block that included an original marker SNP (rs2268388), which corresponded to a part of the *ACACB* gene (Figure 1A and 1B). Therefore, we concluded that *ACACB* was likely a candidate for conferring susceptibility to diabetic nephropathy. We next analyzed 51 SNPs, including 31 tagging SNPs, within *ACACB* in our Japanese population (Japanese1). Several SNPs within the same LD block as rs2268388 were nominally associated with diabetic nephropathy (Figure 1C, Table S1). No single SNP or haplotype showed stronger association with diabetic nephropathy than the original marker SNP (Figure S1).

To validate the association of this SNP with diabetic nephropathy, we examined the effects of the SNP on susceptibility to the disease in several independent populations from different ethnic groups (Table 2). The results indicated that the frequency of the T allele of rs2268388 was consistently higher among patients with type 2 diabetes with proteinuria (combined meta-analysis gave a p value of 5.35×10^−8^ in the Japanese, 2.34×10^−7^ for all populations). Significant association with ESRD was detected in the relatively large European 2 samples (481 cases and 427 controls). The SNP was also modestly associated with ESRD in East Asian type 2 diabetes, but the direction of association differed. Overall, the distribution of the genotype for rs2268388 did not differ significantly between patients with ESRD and control patients having type 2 diabetes (p = 0.47). No significant association was detected in patients with type 1 diabetes having proteinuria.

**Table 2:** Association of the SNP in the *ACACB* (rs2268388) with diabetic nephropathy in several independent cohorts.

Proteinuria				
Populations (a)	Case (b)	Control (b)	*p* (c)	OR (95%CI)
Japanese 1	0.25 (413/276/48)	0.17 (379/155/18)	1.4×10^−6^	1.61 (1.33–1.96)
Japanese 2	0.24 (18/11/2)	0.21 (106/47/10)	0.54	1.23 (0.65–2.34)
Japanese 3	0.29 (35/28/6)	0.22 (116/53/13)	0.09	1.49 (0.96–2.32)
**Japanese meta-analysis** (d)			5.35×10^−8^	1.61 (1.35–1.91)
Heterogeneity test			0.64	
Singaporean Han Chinese	0.25 (108/83/8)	0.24 (119/86/7)	0.64	1.07 (0.78–1.48)
**East Asian meta-analysis** (d)			5.54×10^−7^	1.47 (1.26–1.71)
Heterogeneity test			0.12	
European 1 (Steno 2 study)	0.23 (28/16/3)	0.17 (75/29/4)	0.2	1.48 (0.82–2.68)
**Total meta-analysis** (d)			2.34×10^−7^	1.47 (1.27–1.70)
Heterogeneity test			0.22	
**Total meta-analysis** (d) **excluding Japanese 1**			0.02	1.29 (1.03 – 1.62)
Heterogeneity test			0.30	

ESRD				
Populations	Case	Control	*p* ^b^	OR (95%CI)
Japanese 4	0.19 (196/90/12)	0.23 (136/67/18)	0.12	0.78 (0.58–1.05)
Korean	0.21 (110/58/9)	0.25 (110/75/11)	0.15	0.83 (0.59 – 1.17)
**East Asian meta-analysis** (d)			0.05	0.80 (0.64–1.00)
Heterogeneity test			0.78	
European 2 (Wake Forest)	0.17 (330/128/15)	0.11 (326/86/3)	0.0006	1.61 (1.22–2.13)
**Total meta-analysis** (d)			0.47	1.07 (0.90–1.27)
Heterogeneity test			0.0006	

Type 1 Diabetes				
Populations	Case	Control	*p* ^b^	OR (95%CI)
Dane	0.17 (309/128/11)	0.15 (306/110/9)	0.33	1.13 (0.88–1.47)

**^(a)^**Japanese 1; case-control for a genome-wide association study.

**^(b)^**risk allele frequencies are shown, and genotype counts are in parenthesis.

**^(c)^** *p* values for additive model.

**^(d)^**combined results by using Mantel-Haenszel test.

**Figure 2:**
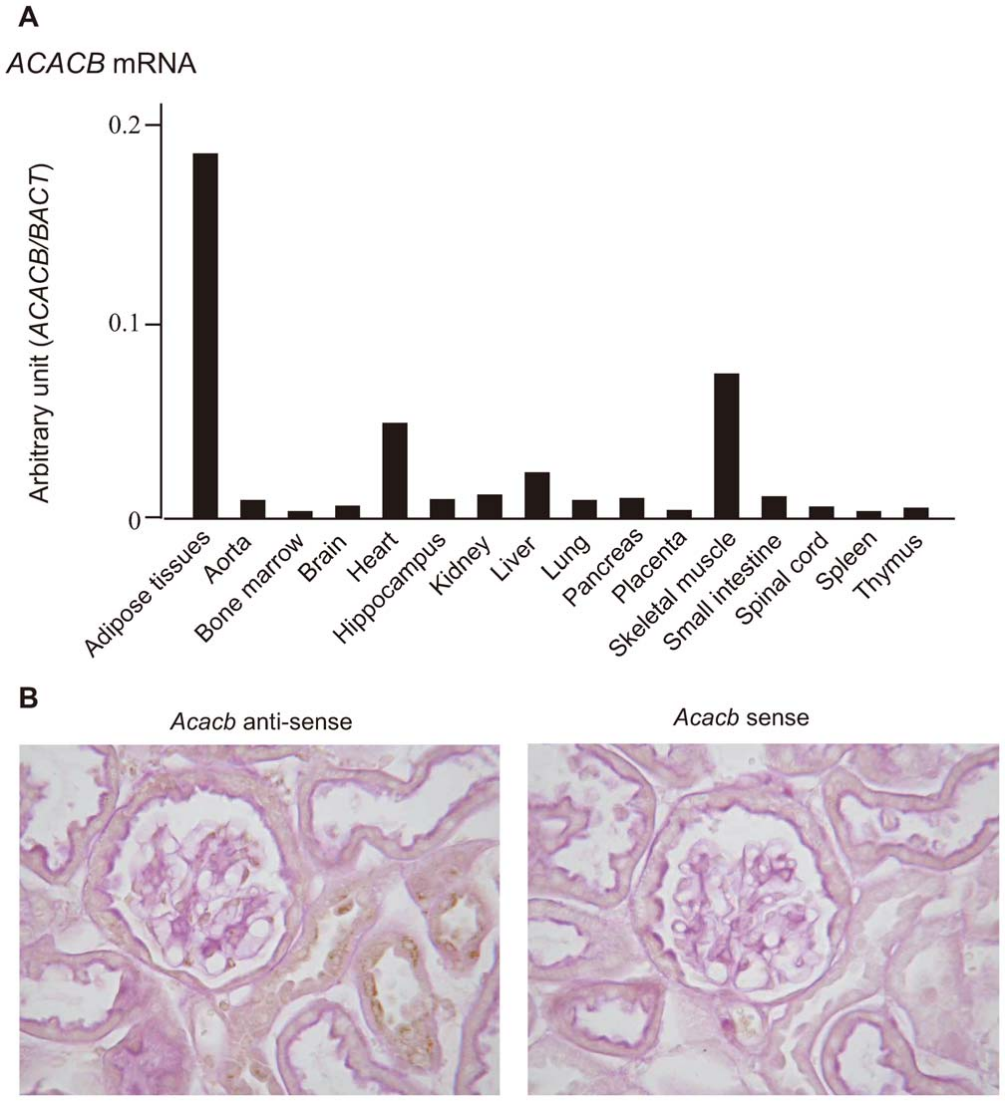
Expression profiles of *ACACB*. (A) Expression profiles of *ACACB* in various human tissues evaluated by real-time PCR. (B) Results of *in situ* hybridization for 20-week-old normal mouse kidneys using mouse *Acacb* anti-sense (left) and sense (right) probes.

We next examined the expression profile of *ACACB* in various human tissues. Expression of *ACACB* was observed in adipose tissue, heart and skeletal muscle, and, to a lesser extent, in the kidney (Figure 2A). The results of *in situ* hybridization with normal mouse kidney revealed that *Acacb* was localized to glomerular epithelial cells and tubular epithelial cells (Figure 2B). We also observed the expression of *ACACB* in cultured human renal proximal tubular epithelial cells (hRPTECs).

**Figure 3:**
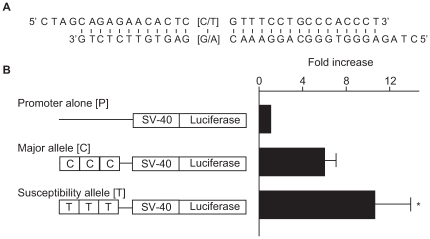
Effect of a 29-bp DNA fragment containing the associated SNP (rs2268388) on transcriptional activity in cultured hRPTECs. (A) A 29-bp sequence including SNP rs2268338. Overhung sequences are compatible ends for concatenation. (B) Enhancer activities of the DNA fragments corresponding to each allele in human RPTECs. Results are shown as mean ± SD of the ratio of activity to that of the promoter alone obtained from 4 independent experiments. * p = 0.045 vs. major allele.

To investigate the functional role of this SNP region, we examined the effects of a 29-bp DNA fragment containing the associated SNP (rs2268388) on transcriptional activity in cultured hRPTECs. As shown in the Figure 3, the 29-bp DNA fragments had significant enhancer activity (promoter alone [P]: 39.4±13.1; susceptibility allele [T]: 384.3±104.1; major allele [C]: 238.5±81.9; relative luciferase activity, p = 0.0005 for P vs. T, p = 0.016 for P vs. C). Fragments corresponding to the disease susceptibility allele had stronger enhancer activity than those for the major allele ([T] 10.5±3.4 vs. [C] 5.9±1.3, fold increase over promoter alone, p = 0.045, Figure 3B).

## Discussion

In the present study, we showed *ACACB* located at chromosome 12q24.1 to be a strong susceptibility gene for diabetic nephropathy in patients with type 2 diabetes. Our findings suggest that a SNP within *ACACB* (rs2268388, intron 18 + 4139 C > T) contributes to the development of proteinuria in patients with type 2 diabetes.

*ACACB* encodes acetyl-coenzyme A (CoA) carboxylase beta, which catalyzes the carboxylation of acetyl-CoA to malonyl-CoA, and controls fatty acid oxidation by means of the ability of malonyl-CoA to inhibit carnitine palmitoyl transferase I (*CPT1A*; MIM 600528), the rate-limiting step in fatty acid uptake and oxidation by mitochondria in non-lipogenic tissues. Mice lacking *Acacb* have a normal life span, a higher rate of fatty acid oxidation, lower amounts of fat, and increased insulin sensitivity [19]–[21]; therefore, *ACACB* might affect insulin sensitivity via modulation of fatty acid metabolism. However evidence suggesting a role for the *ACACB* in the pathogenesis of diabetic nephropathy was previously lacking. In this study, expression of *ACACB* was detected in heart, skeletal muscle and adipose tissues by real-time quantitative polymerase chain reaction (PCR) as previously reported [22]. We also showed that *ACACB* was expressed in human kidney, and *in situ* hybridization revealed that *Acacb* expression was localized to glomerular epithelial cells and tubular epithelial cells in normal mouse kidneys. Abnormalities in lipid metabolism [23],[24], including fatty acid metabolism have been shown to contribute to the development and/or progression of chronic kidney diseases, including diabetic nephropathy. Hence, genotype-based differences in expression and/or activity of this enzyme in the kidney might contribute to conferring susceptibility to diabetic nephropathy.

Elucidating these functional differences will help us to understand how variation in this gene contributes to susceptibility to diabetic nephropathy. In this study, the 29-bp fragment that included the landmark SNP (rs2268388) was shown to have significant enhancer activity. We also demonstrated that the DNA corresponding to the disease susceptibility allele had significantly higher enhancer activity than that for the major allele in cultured human RPTECs. Therefore, the intronic variation in the gene seems to be causal. We hypothesize that higher expression of *ACACB* in the kidneys of subjects having the disease susceptibility allele (T) may increase the susceptibility to diabetic nephropathy in type 2 diabetes.

Interestingly, the association of the T allele of rs2268388 with type 2 diabetes-associated nephropathy was consistently observed in cases with proteinuria, whereas there was less consistent association between type 2 diabetic patients under chronic renal replacement therapy (ESRD). Discrepancies in genomic loci underlying susceptibility to proteinuria versus ESRD were previously noted in a genome-wide linkage scan for diabetic nephropathy in type 2 diabetes [25]. Because most of the patients with ESRD were considered to have had proteinuria, and there is significant heterogeneity in the association with diabetic ESRD among the East Asian and European American populations (heterogeneity p = 0.0006, Table 2), some selection bias, such as a survival effect, or ethnic differences might exist when patients with ESRD were used as cases. Since the presence of proteinuria is also recognized as a predictor of cardio-vascular diseases, the association of *ACACB* with proteinuria might reflect an association between the gene and cardio-vascular diseases or metabolic syndrome. However elucidation of a precise mechanism will require further investigation.

Association of the *ACACB* with diabetic nephropathy could not be replicated in patients with type 1 diabetes, although these nephropathy cases had proteinuria. The clinical features or histological characteristics of diabetic nephropathy are similar in both type1 and type 2 diabetes mellitus; however, there are some differences in the background circumstances between both types of the disease. For example, patients with type 2 diabetes are generally older, and more obese than those with type 1 diabetes. Therefore, it is possible that genetic factors for nephropathy are different for type 1 and type 2 diabetes, although some overlap may exist. Because the statistical power of this study for patients with type 1 diabetes was probably not sufficient, the association of variations in *ACACB* with diabetic nephropathy in patients with type 1 diabetes should be re-evaluated in future studies.

Recently, GWASs have been performed to identify susceptibility genes for common diseases. Convincing susceptibility genes for many diseases including type 2 diabetes have been successfully identified by GWASs. A GWAS for diabetic nephropathy was conducted in the Genetics of Kidneys in Diabetes (GoKinD) collection, and several novel loci were identified for diabetic nephropathy in type 1 diabetes [11]. Compared to our study, recent GWASs conducted in European and American groups had greater power to detect true associations. The power of the first and second test in the present study is estimated >40% and >90% respectively, for SNPs with minor allele frequency of 0.2 as in our sample, if we set a cut-off value at the p = 0.05 level, a genotypic relative risk (γ) of 1.5, and the prevalence of diabetic nephropathy is assumed to be 10% (CaTS power calculator, CaTS: http://www.sph.umich.edu/csg/abecasis/CaTS/). Therefore, other important loci contributing to diabetic nephropathy in Japanese populations may not have been detected and should be searched for using a larger scale of GWAS. Limitations exist in increasing the numbers of subjects with diabetic nephropathy and diabetes lacking nephropathy in single center studies. Therefore, we examined several independently collected study populations, allowing for the presence of different selection criteria and the small sample size in some replication cohorts. Future analyses should attempt to avoid these limitations so as not to produce spurious results.

In summary, based upon extensive genome-wide gene-centric SNP analyses, we identified *ACACB* as a candidate gene for conferring susceptibility to diabetic nephropathy. Subsequent replication studies in several different ethnic groups and a functional study suggest that the T-allele of a common intronic SNP (rs2268338) within *ACACB* is a risk factor for the development and progression of proteinuria in patients with type 2 diabetes.

## Materials and Methods

### Subjects, DNA preparation, and genotyping

Japanese 1 (genome-wide screening); DNA samples were obtained from the peripheral blood of patients with type 2 diabetes who regularly attended outpatient clinics at Shiga University of Medical Science, Tokyo Women's Medical University, Juntendo University, Kawasaki Medical School, Iwate Medical University, Toride Kyodo Hospital, Kawai Clinic, Osaka City General Hospital, Chiba Tokusyukai Hospital or Osaka Rosai Hospital. All subjects provided informed consent before enrolling in this study. DNA extraction was performed by a standard phenol-chloroform method. Diabetic patients were divided into 2 groups according to the following diagnostic criteria: 1) nephropathy cases, i.e., patients with diabetic retinopathy and overt nephropathy, indicated by a urinary albumin excretion rate (AER) ≥200 µg/min or a urinary albumin/creatinine ratio (ACR) ≥300 mg/g creatinine (Cr), and 2) control patients who have diabetic retinopathy but no evidence of renal dysfunction (i.e. AER less than 20 µg/min or ACR less than 30 mg/g Cr). Measurements of AER or ACR were performed at least twice for each patient. The SNPs for genotyping were randomly selected from our gene-based Japanese SNP (JSNP) database. The genotype of each SNP locus was analyzed with multiplex PCR-invader assays, as described previously [14]–[16].

Our first screening involved genotyping 94 nephropathy cases and 94 control patients for more than 100,000 SNP loci. In total, 76,767 SNP loci, which distributed to 13,707 gene-centric regions and covered approximately 35% of common SNPs (MAF >0.15) in these regions, were successfully genotyped by the invader assay (success rates >0.95). We estimated identity by descent (IBD) sharing to assess relatedness among our initial GWAS populations. As shown in Figure S2, the result indicates that there were no close relative pairs in this population. We also performed principal component analysis (PCA) using our initial GWAS populations to evaluate population structures. Since all subjects were in a single cluster in the PCA analysis (Figure S3), no evidence for population stratification between case and control groups appeared to exist in the present GWAS.

After the first round of analysis that evaluated SNPs with p values less than 0.01 in the first screening (previously published [14]–[16]), we extended our analysis to include SNPs with p values between 0.01 and 0.05 and examined them in a larger number of patients (754 nephropathy cases vs. 558 control patients). The study protocol was approved by the ethics committees of RIKEN Yokohama Institute and each participating institution.

### Subjects in replication studies

#### Japanese replication studies

We obtained DNA samples from 2 longitudinal studies (Japanese 2, Japanese 3) and one case-control study (Japanese 4).

Japanese 2; Patients with type 2 diabetes were recruited from among the participants in the Shiga Prospective Observational Follow-Up Study for Diabetic Complications [26]. On the basis of at least 2 measurements of AER in 24-h urine collections, those classified as having microalbuminuria (200 µg/min > AER ≥20 µg/min) were followed for up to 6 years. The progressors (cases) were defined as those had progressed to overt proteinuria (AER ≥200 µg/min, n = 32), and the remaining patients were defined as non-progressors (controls, n = 168). The *ACACB* genotype was analyzed with multiplex PCR-invader assays. The study protocol and informed consent procedure were approved by the ethics committee of Shiga University of Medical Science.

Japanese 3; Patients with type 2 diabetes and normoalbuminuria or microalbuminuria, as determined by at least two measurements of ACR or AER (normoalbuminuria; ACR <30 mg/g Cr or AER <20 µg/min, microalbuminuria; 30≤ ACR <300 mg/g Cr or 20≤ AER <200 µg/min) who could be followed for 10 years were recruited from among diabetic outpatients at Juntendo University Hospital or Saiseikai Central Hospital [27]. Progressors (cases, n = 71) were defined as patients who progressed from a given stage to a more advanced stage of diabetic nephropathy; the remaining patients were defined as non-progressors (controls, n = 193). The *ACACB* genotype was analyzed with multiplex PCR-invader assays. All patients gave informed consent and the protocol was approved by the ethics committee of Juntendo University or that of Saiseikai Central Hospital.

Japanese 4; Patients with type 2 diabetes regularly visiting Tokai University Hospital or its affiliated hospitals were enrolled. All nephropathy cases were receiving chronic hemodialysis therapy (n = 300), and control patients were those with normoalbuminuria determined by at least 2 measurements of the urinary ACR, and diabetes for more than 10 years (n = 218). The *ACACB* genotype was analyzed with multiplex PCR-invader assays. Patients gave informed consent and the protocol was approved by the ethics committee of Tokai University School of Medicine.

#### Korean replication study

Korean patients with type 2 diabetes comprising two groups according to the following criteria were examined [28]: 1) the control group (n = 196): patients with diabetic retinopathy and who had diabetes for more than 15 years but no renal involvement (i.e., ACR<30 mg/g Cr and creatinine clearance [using the Cockroft equation] of >60 ml/min per 1.73 m^2^); 2) the ESRD group (n = 177): patients with diabetic retinopathy and ESRD due to type 2 diabetes, as indicated by a creatinine clearance rate of <15 ml/min per 1.73 m^2^ or receiving renal replacement therapy. The TaqMan method for genotyping was applied in the Korean replication study. The institutional review board of the Clinical Research Institute at Seoul National University Hospital approved the study protocol, and informed consent for genetic analysis was obtained from each patient.

#### Singaporean replication study

Cases and controls were selected from Chinese patients with type 2 diabetes who had been enrolled into the Singapore Diabetes Cohort Study (SDCS) as previously reported [29]. Patients with ACR >300 mg/g Cr or dipstick positive were considered nephropathy cases (n = 199). Controls were patients who were normalbuminuric with ACR <30 mg/g Cr and had diabetes for more than 7 years (n = 212). The *ACACB* genotype was analyzed using a Taqman genotyping assay available from Applied Biosystems (Foster city, CA, U.S.A.). The research protocol for SDCS was approved by both the National University of Singapore Institutional Review Board (NUS-012) and the National Healthcare Group Domain-Specific Review Board (C/05/118).

#### European replication studies

European 1 (Steno 2): Patients were recruited from the Steno Diabetes Center between 1992 and 1993. Microalbuminuria was defined as an AER of 30–300 mg per 24 h in 4 of 6 samples of sterile urine. These patients were enrolled and followed up for an average of 7.8 years [30]. Patients who progressed to nephropathy (AER >300 mg per 24 h, n = 47) were used as cases, and the remaining patients were defined as controls (n = 110). The *ACACB* genotype was analyzed with multiplex PCR-invader assays. Informed consent was obtained from all participants. The protocol was in accordance with the Declaration of Helsinki and was approved by the ethics committee of Copenhagen County.

European 2 (Wake Forest): Patients with European ancestry who were born in North Carolina, South Carolina, Georgia, Tennessee, or Virginia were enrolled. Cases all had type 2 diabetes mellitus for 5 or more years before the development of ESRD with overt proteinuria and/or diabetic retinopathy (n = 481). Control patients had type 2 diabetes for more than 5 years with ACR <30 mg/g Cr and serum creatinine <1.5 mg/dl (n = 427). The *ACACB* SNP was genotyped using the MassARRAY genotyping system (Sequenom, San Diego, CA, U.S.A.). PCR primers were designed using the MassARRAY Design 3.4 Software (Sequenom). This study was conducted under Institutional Review Board approval from Wake Forest University School of Medicine, and adhered to the tenets of the Declaration of Helsinki.

Type 1 diabetes: Adults with type 1 diabetes attending the outpatient clinic at the Steno Diabetes Center were invited to participate in a study of genetic risk factors for the development of diabetic micro- and macrovascular complications [31]. Patients were considered to have type 1 diabetes if the age at onset of diabetes was ≤35 years and if the time to definitive insulin therapy was ≤1 year. Established diabetic nephropathy (cases, n = 458) was defined as persistent albuminuria (≥300 mg/24 h) in 2 out of 3 consecutive measurements on sterile urine in the presence of retinopathy. The absence of diabetic nephropathy (controls, n = 442) was defined as persistent normoalbuminuria (urinary albumin excretion rate: <30 mg/24 h) after at least 15 years of diabetes duration in patients not treated with angiotensin converting enzyme inhibitors or angiotensin II receptor blockers. The *ACACB* genotype was analyzed with multiplex PCR-invader assays. The study was performed in accordance with the Declaration of Helsinki. The local ethics committee approved the study and all patients gave their informed consent.

The clinical characteristics of patients in all studies are shown in Table S2.

### Real-time quantitative RT–PCR

We obtained human cDNAs from multiple tissues from CLONTECH Inc. (Palo Alto, CA, U.S.A.). The cDNAs were amplified by PCR with the following primers: human *ACACB*, sense 5′-CGG ATG CGT AAC TTC GAT CTG-3′, antisense 5′-CTA TGG TCC GTC ACT TCC ACA C-3′; *BACT*, sense 5′- TCA CCC ACA CTG TGC CCA TCT ACG A -3′, antisense 5′- CAG CGG AAC CGC TCA TTG CCA ATG G -3′. Amplification was performed in a 22 µl reaction volume that contained 1× EX Taq Buffer, 200 nM dNTP, 1/20,000 SYBR Green, 0.2 µM Rox, 800 nM gene-specific primer, 0.05 U/µl EX Taq Hot Start Version (Takara, Otsu, Japan), and 5 ng of template DNA. The thermal profile was 50°C for 2 min, at 95°C for 10 min, followed by 40 cycles at 95°C for 30 s and at 60°C for 60 s in thermal cycler (Mx3000P Multiplex Quantitative PCR system; Stratagene, La Jolla, CA, U.S.A.). The results were normalized with human *BACT*.

### *In situ* hybridization

Under pentobarbital anesthesia, 20-week-old mice were flushed with PBS through the abdominal aorta followed by perfusion with 4% paraformaldehyde buffered with PBS (pH 7.4). The kidneys were quickly removed and cut into small pieces. The renal cortex tissue was immediately dissected and immersed into a fresh portion of the same fixative at 4°C overnight. All steps were carefully carried out to avoid contamination with RNase. Diethylpyrocarbonate-treated water was used at 0.1% to prepare each buffer. The fixed samples were thoroughly rinsed with PBS (pH 7.4) and subsequently dehydrated by passage through an alcohol series and cleared in xylene. *In situ* hybridization was performed on paraffin-embedded sections using a previously described method [15]. Antisense and sense single-strand cRNAs were synthesized from cDNA fragments encoding *Acacb* using reverse-transcription PCR. The *Acacb* cDNA fragment was consisted of a 500 bp mouse sequence (nucleotides 181–680, GenBank accession number NM_133904, GenBank: http://www.ncbi.nlm.nih.gov/Genbank/).

### Plasmid construction and transfection experiments

Three copies of the 29-bp DNA fragments including rs2268388 in *ACACB* were subcloned into a pGL3-promoter vector (Promega, Madison, WI, U.S.A.) at its multi-cloning site upstream of the SV-40 promoter. We introduced constructs corresponding to each allele into the human renal proximal tubular epithelial cells (hRPTEC, Lonza, Basel, Switzerland) along with a sea-pansy luciferase control vector, pRL-TK (Promega), using the liposome transfection procedure (Lipofectoamine 2000, Life Technology Inc, Carlsbad, CA, U.S.A.). Twenty-four hours after transfection, luciferase activitiy was determined by means of the Dual Luciferase Reporter Assay System (Promega). The luminescence of firefly luciferase was corrected by use of the sea-pansy luciferase, which reflected transfection efficiency.

### Statistical analyses

We tested the genotype and allele frequencies for Hardy-Weinberg equilibrium (HWE) proportions by use of the χ^2^ test [32]. We calculated the LD index, D' and r^2^, as described elsewhere [33]. We analyzed the differences between the case and control groups with regard to the genotype distribution and allele frequency in the genome-wide screen by Fisher's exact test with dominant, recessive and allelic models with autosomal SNPs. The association of the *ACACB* locus with diabetic nephropathy in the replication study was evaluated with the Armitage test for trends using an additive model, as described previously [34]. Combined meta-analysis was performed by using the Mantel-Haenszel procedure with a fixed effect model after testing for heterogeneity. The data from the transfection experiments were analyzed by one-way analysis of variance, followed by Scheffe's test to evaluate statistical differences among 3 groups or by an un-paired t test to evaluate differences between 2 groups.

## Supporting Information

Figure S1 Haplotype frequencies and the association of haplotypes in the ACACB with diabetic nephropathy. Fifty-one SNPs within the ACACB were genotyped in 754 nephropathy cases and 558 controls. Nine haplotype blocks are identified using Gabriel's Method by Haploview.
(0.58 MB EPS)

Figure S2 Estimation of IBD sharing among subjects in the genome-wide screening.
(0.24 MB EPS)

Figure S3 Quality control for the genome-wide screening. (A) Principal Compornent Analysis (PCA) (B) quantile-quantile plot.
(0.42 MB EPS)

Table S1 Association of SNPs in the ACACB gene with diabetic nephropathy in Japanese subjects with type 2 diabetes. (A) tagging SNPs to cover this locus are shown in bold. (B) Minor allele frequencies are presented. Genotype counts are in parenthesis. 11; homozygous for major allele, 12; heterozygous, 22; homozygous for minor allele. (C) p values for the additive model.
(0.14 MB DOC)

Table S2 Clinical characteristics of the subjects. Values are mean ± SE, NA: not available. (A) Data at baseline are presented. (B) 5 unknown. (C) 6 unknown. (D) p<0.05 versus control.
(0.09 MB DOC)
