# Supplementary material for: A Single Nucleotide Polymorphism within the Acetyl-Coenzyme A Carboxylase Beta Gene Is Associated with Proteinuria in Patients with Type 2 Diabetes
Source: PLoS Genet. 2010 Feb 12;6(2):e1000842. doi: 10.1371/journal.pgen.1000842 (PMC2820513; doi:10.1371/journal.pgen.1000842)
Supplement: Table S1 — Association of SNPs in the ACACB gene with diabetic nephropathy in Japanese subjects with type 2 diabetes. (A) tagging SNPs to cover this locus are shown in bold. (B) Minor allele frequencies are presented. Genotype counts are in parenthesis. 11; homozygous for major allele, 12; heterozygous, 22; homozygous for minor allele. (C) p values for the additive model. (0.14 MB DOC) [file pgen.1000842.s004.doc]

| SNPs ^(a)^ | Position in the chromosome | Nephropathy^(b)^  (11/12/22) | Control^(b)^  (11/12/22) | *P*^(c)^ | Odds ratio  (95% CI) |
| --- | --- | --- | --- | --- | --- |
| rs1654873 (C > T) | 108064588 | 0.36  (308/335/98) | 0.37  (219/254/78) | 0.45 |  |
| rs1642040 (C > A) | 108065343 | 0.37  (284/326/105) | 0.38  (184/237/63) | 0.92 |  |
| **rs2430683 (T > G)** | 108066588 | 0.15  (531/167/25) | 0.16  (343/132/13) | 0.44 |  |
| **rs3753166 (A > G)** | 108067358 | 0.22  (435/243/35) | 0.21  (295/165/20) | 0.73 |  |
| **rs246090 (G > T)** | 108074044 | 0.36  (299/327/98) | 0.38  (187/233/68) | 0.40 |  |
| **rs34266 (T > C)** | 108080234 | 0.37  (286/334/102) | 0.38  (185/230/70) | 0.66 |  |
| **rs7299500 (C > T)** | 108082939 | 0.25  (412/267/47) | 0.26  (266/189/34) | 0.43 |  |
| **rs12426388 (T > C)** | 108084048 | 0.26  (397/265/49) | 0.26  (265/175/39) | 0.64 |  |
| rs7976552 (A > G) | 108084958 | 0.30  (362/290/68) | 0.29  (251/193/45) | 0.78 |  |
| rs34286 (G > A) | 108090379 | 0.24  (413/262/45) | 0.26  (276/176/37) | 0.51 |  |
| rs246092 (G > A) | 108091434 | 0.29  (240/183/44) | 0.31  (217/202/44) | 0.28 |  |
| **rs246095 (G > T)** | 108091934 | 0.25  (415/283/45) | 0.25  (319/197/34) | 0.56 |  |
| **rs2284697 C > T)** | 108092105 | 0.49  (173/313/159) | 0.49  (117/225/112) | 0.81 |  |
| **rs7301145 (G > C)** | 108096723 | 0.30  (351/303/66) | 0.33  (224/203/57) | 0.19 |  |
| rs2284695 (A > G) | 108100366 | 0.30  (223/202/40) | 0.33  (207/199/54) | 0.16 |  |
| **rs2268404 (G > A)** | 108101239 | 0.05  (679/64/3) | 0.06  (482/65/3) | 0.05 |  |
| rs10849921 (C > G) | 108106623 | 0.21  (460/222/40) | 0.23  (292/166/31) | 0.15 |  |
| **rs2300456 (C > T)** | 108107477 | 0.12  (553/148/13) | 0.13  (386/92/17) | 0.17 |  |
| **rs2300455 (C > T)** | 108107899 | 0.27  (377/291/51) | 0.24  (277/180/27) | 0.08 |  |
| rs1016331 (C > T) | 108108679 | 0.31  (223/200/42) | 0.34  (204/196/55) | 0.16 |  |
| rs2268403 (G > A) | 108109090 | 0.41  (257/362/125) | 0.39  (207/259/85) | 0.23 |  |
| rs2268400 (G > A) | 108111160 | 0.18  (500/214/30) | 0.21  (340/189/23) | 0.06 |  |
| rs2287221 (C > T) | 108114378 | 0.25  (413/284/46) | 0.24  (316/200/32) | 0.48 |  |
| **rs2268392 (A > G)** | 108119459 | 0.35  (279/291/80) | 0.34  (196/213/48) | 0.66 |  |
| **rs2268390 (G > A)** | 108119660 | 0.21  (473/232/40) | 0.24  (317/202/32) | 0.06 |  |
| **rs4766455 (G > C)** | 108125961 | 0.42  (240/353/124) | 0.40  (179/229/79) | 0.29 |  |
| **rs2268389 (T > C)** | 108127535 | 0.19  (494/220/31) | 0.19  (358/169/18) | 0.94 |  |
| **rs2268388 (C > T)** | 108128028 | 0.25  (413/276/48) | 0.17  (379/155/18) | 1.4 × 10^-6^ | 1.61  (1.33 – 1.96) |
| **rs2268387 (T > C)** | 108128078 | 0.26  (384/286/46) | 0.26  (264/187/30) | 0.69 |  |
| rs2239608 (T > G) | 108131105 | 0.34  (327/334/84) | 0.27  (283/232/33) | 0.0005 | 1.36  (1.15 – 1.61) |
| rs2239607 (T > C) | 108131663 | 0.29  (234/196/39) | 0.24  (259/184/21) | 0.016 | 1.28  (1.04 – 1.57) |
| **rs2300452 (C > T)** | 108142453 | 0.29  (367/313/61) | 0.24  (311/214/25) | 0.002 | 1.31  (1.10 – 1.57) |
| rs2075258 (T > C) | 108146362 | 0.34  (327/330/85) | 0.28  (284/233/36) | 0.0008 | 1.33  (1.13 – 1.58) |
| **rs9971877 (T > C)** | 108154053 | 0.36  (302/323/95) | 0.32  (220/224/44) | 0.06 |  |
| **rs3742027 (G > A)** | 108154851 | 0.37  (288/363/95) | 0.42  (185/258/103) | 0.005 | 0.80  (0.68 – 0.93) |
| **rs3742026 (C > G)** | 108155040 | 0.29  (366/325/52) | 0.29  (268/237/42) | 0.79 |  |
| **rs2241220 (C > T)** | 108159412 | 0.30  (355/304/61) | 0.35  (210/212/61) | 0.01 | 0.79  (0.67 – 0.95) |
| **rs2300451 (T > C)** | 108164483 | 0.06  (657/80/4) | 0.09  (452/92/5) | 0.001 | 0.62  (0.46 – 0.83) |
| rs759560 (C > T) | 108167442 | 0.31  (221/207/39) | 0.35  (196/211/56) | 0.04 | 0.82  (0.68 – 0.10) |
| **rs4766587 (G > A)** | 108169713 | 0.35  (313/313/92) | 0.37  (190/234/61) | 0.29 |  |
| **rs2284689 (C > T)** | 108169928 | 0.21  (464/246/32) | 0.20  (350/172/26) | 0.78 |  |
| rs2284686 (G > A) | 108171104 | 0.22  (452/257/34) | 0.22  (343/178/31) | 0.79 |  |
| **rs2284685 (C > G)** | 108171165 | 0.36  (297/350/96) | 0.35  (229/258/63) | 0.41 |  |
| rs3742025 (C > T) | 108172194 | 0.002  (648/2/0) | 0.001  (458/1/0) | 0.78 |  |
| **rs2066925 (A > T)** | 108174697 | 0.31  (364/303/76) | 0.30  (275/222/55) | 0.77 |  |
| **rs2075259 (G > A)** | 108176374 | 0.27  (387/295/54) | 0.31  (266/228/55) | 0.06 |  |
| **rs3742023 (G > A)** | 108178365 | 0.39  (241/312/97) | 0.38  (171/221/62) | 0.66 |  |
| rs10849968 (C > A) | 108178703 | 0.30  (355/290/71) | 0.29  (248/198/45) | 0.61 |  |
| rs2075260 (A > G) | 108181221 | 0.24  (435/261/48) | 0.23  (332/188/34) | 0.55 |  |
| **rs2075262 (G > A)** | 108188560 | 0.47  (198/394/153) | 0.49  (148/266/136) | 0.33 |  |
| rs2075263 (T > C) | 108188708 | 0.23  (444/259/43) | 0.23  (333/181/34) | 0.75 |  |
