# Supplementary material for: A Single Nucleotide Polymorphism within the Acetyl-Coenzyme A Carboxylase Beta Gene Is Associated with Proteinuria in Patients with Type 2 Diabetes
Source: PLoS Genet. 2010 Feb 12;6(2):e1000842. doi: 10.1371/journal.pgen.1000842 (PMC2820513; doi:10.1371/journal.pgen.1000842)
Supplement: Table S2 — Clinical characteristics of the subjects. Values are mean ± SE, NA: not available. (A) Data at baseline are presented. (B) 5 unknown. (C) 6 unknown. (D) p<0.05 versus control. (0.09 MB DOC) [file pgen.1000842.s005.doc]

|  | Japanese 1 | | Japanese 2 ^a^ | | Japanese 3 ^a^ | |
| --- | --- | --- | --- | --- | --- | --- |
|  | Case  proteinuria | control | Progression  proteinuria | control | Progression  proteinuria | control |
| Sex (M:F) | 505:244^(b) (d)^ | 267:285^(c)^ | 20:12 | 110:58 | 53:18 | 124:69 |
| Age (year) | 60.1 ± 0.4^(d)^ | 62.4 ± 0.5 | 60.9 ± 1.7 | 60.4 ± 0.7 | 53.6 ± 1.3 | 53.1 ± 0.8 |
| BMI (kg/m^2^) | 23.7 ± 0.2 | 23.6 ± 0.2 | 24.9 ± 0.5 | 23.9 ± 0.3 | 22.3 ± 0.4^(d)^ | 23.9 ± 0.2 |
| HbA1c (%) | 7.6 ± 0.2 | 7.6 ± 0.1 | 7.7 ± 0.2 | 7.4 ± 0.1 | 8.9 ± 0.3^(d)^ | 7.8 ± 0.1 |
| SBP (mmHg) | 140 ± 1^(d)^ | 132 ± 1 | 138 ± 3 | 137 ± 1 | 128 ± 2 | 130 ± 2 |
| DBP (mmHg) | 76 ± 0.5^(d)^ | 74 ± 0.5 | 77 ± 2 | 77 ± 1 | 77 ± 1 | 76 ± 2 |
| Duration (year) | 19.3 ± 0.4^(d)^ | 15.3 ± 0.4 | 14.5 ± 1.6 | 12.8 ± 0.7 | 8.5 ± 0.9 | 7.6 ± 0.6 |

|  | Japanese 4 | | Korean | | Singapore | |
| --- | --- | --- | --- | --- | --- | --- |
|  | case  ESRD | control | case  ESRD | control | Case  Proteinuria | control |
| Sex (M:F) | 199 : 101 | 149 : 69 | 105:72^(d)^ | 76:120 | 112:87^(d)^ | 87:125 |
| Age (year) | 64.4 ± 0.6 | 65.0 ± 0.7 | 61.0 ± 0.7^(d)^ | 63.8 ± 0.6 | 64.9 ± 0.7 | 64.8 ± 0.6 |
| BMI (kg/m^2^) | 22.1 ± 0.2^(d)^ | 23.4 ± 0.3 | 23.2 ± 0.2 | 23.7 ± 0.2 | 25.7 ± 0.3^(d)^ | 24.7 ± 0.3 |
| HbA1c (%) | 6.12 ± 0.07^(d)^ | 6.80 ± 0.07 | 7.2 ± 0.1^(d)^ | 7.8 ± 0.1 | 7.8 ± 0.1^(d)^ | 7.5 ± 0.1 |
| SBP (mmHg) | 142 ± 1^(d)^ | 121 ± 1 | 150 ± 2^(d)^ | 131 ± 1 | 137 ± 1^(d)^ | 135 ± 1 |
| DBP (mmHg) | 73 ± 1^(d)^ | 69 ± 1 | 83 ± 1^(d)^ | 77 ± 1 | 78 ± 1^(d)^ | 76 ± 1 |
| Duration (year) | 21.9 ± 0.9^(d)^ | 16.3 ± 0.4 | 18.1 ± 0.6^(d)^ | 20.6 ± 0.4 | 11.2 ± 1.0^(d)^ | 17.7 ± 1.0 |

|  | European 1 (Steno 2) ^a^ | | European 2 (Wake Forest) | | Steno Type 1 | |
| --- | --- | --- | --- | --- | --- | --- |
|  | Progression  proteinuria | control | case  ESRD with ever proteinuria | control | case  proteinuria | control |
| Sex (M:F) | 37:10 | 80:30 | 235:246 | 188:239 | 278:180^(d)^ | 234:208 |
| Age (year) | 57.2 ± 1.0^(d)^ | 54.1 ± 0.7 | 65.5 ± 0.5^(d)^ | 63.1 ± 0.4 | 42.1 ± 0.5^(d)^ | 45.4 ± 0.5 |
| BMI (kg/m^2^) | 29.2 ± 0.6 | 30.1±0.4 | 29.4 ± 0.3^(d)^ | 32.5 ± 0.3 | 24.2 ± 0.2 | 24.2 ± 0.1 |
| HbA1c (%) | 8.4 ± 0.2 | 8.6 ± 0.2 | NA | NA | 9.4 ± 0.1^(d)^ | 8.4 ± 0.1 |
| SBP (mmHg) | 150 ± 3 | 147 ± 2 | NA | NA | 144 ± 1^(d)^ | 134 ± 1 |
| DBP (mmHg) | 86 ± 2^(d)^ | 85 ± 1 | NA | NA | 82 ± 1^(d)^ | 76 ± 1 |
| Duration (year) | 7.8 ± 0.8 | 6.8 ± 0.6 | 21.0 ± 0.4^(d)^ | 12.7 ± 0.4 | 28.2 ± 0.4 | 27.8 ± 0.5 |
